# Effects of Thioglycolate Compounds in an Emerging Technique in the World of Cosmetics—Brow Lamination

**DOI:** 10.1111/jocd.16654

**Published:** 2024-10-29

**Authors:** Laura Ghanem, Stephani Chagoury, Andrea Issa, Kaline Maya Khoury, Kelly Katherine Karam, Milissa Makhlouf

**Affiliations:** ^1^ Faculty of Medical Sciences Lebanese University Beirut Lebanon; ^2^ Faculty of Medicine University of Balamand Tripoli Lebanon

**Keywords:** ammonium thioglycolate, brow lamination, chemicals, eyebrows, skin irritation, thioglycolic acid

## Abstract

**Background:**

The side effects of two related chemicals, ammonium thioglycolate (ATG) and thioglycolic acid (TGA) have been widely highlighted in the world of cosmetics. These thioglycolate compounds are considered essential ingredients in a new technique known as brow lamination. This technique is widely used nowadays, with the aim of changing the eyebrow shape.

**Aims:**

To our knowledge, this is the first study to address the possible side effects of brow lamination.

**Results:**

The hydrophilic characteristic of ATG and TGA reflects their transdermal absorption through the intracellular and transappendageal pathways. These compounds can affect the skin through allergic contact dermatitis (ACD), characterized by skin irritation, dryness, and erythema. Moreover, these thioglycolates can alter several mechanical and chemical reactions in the eyebrows' hair, therefore affecting their shape, structure, and pigmentation. In addition, these chemicals contained in brow lamination can exert systemic manifestations, at the level of the reproductive, ocular, respiratory, and endocrine systems.

**Conclusion:**

More studies should be elaborated to shed light on the possible side effects of this trend. Additionally, further regulations should be taken into consideration to ensure the concentration and the measures applied are convenient to minimize these side effects.

**Abbreviations**
1O2: singlet oxygen
βHB: β‐hydroxybutyrate
AA: alopecia areata
ACD: allergic contact dermatitis
APCs: antigen‐presenting cells
ATG: ammonium thioglycolate
CCCA: central centrifugal cicatricial alopecia
ETC: electron transport chain
GGG: gauche‐gauche‐gauche
GGT: gauche‐gauche‐trans
GM‐CSF: granulocyte‐macrophage colony‐stimulating factor
IL: interleukin
MHC: major histocompatibility complex
NADPH: nicotinamide adenine dinucleotide phosphate
O2^−^
: superoxide radicals
ORS: outer root sheath
OSI: oxidative stress index
SS–: disulfide
TAC: total antioxidant capacity
TGA: thioglycolic acid
Th1: T helper type 1
Th17: T helper type 17
Th2: T helper type 2
TNF‐α: tumor necrosis factor‐α
TOC: total oxidant capacity
UVA: ultraviolet A
UVB: ultraviolet B.

## 1 Introduction

The eyebrows are small tilted hairs [1]. Their form and location above the eyes shield the eyes from light, sweat, and dust [1]. Throughout history, eyebrow shaping has seen substantial alterations because of beauty trends that dictate different forms and styles based on racial, gender, and social criteria [2]. Women seeking to improve their look through cosmetic surgery have resorted to procedures including multidirectional thread lifting, eyebrow transplantation, and many more [2, 3, 4]. Yet, because they are less invasive, semi‐permanent procedures like microblading and brow lamination are trending nowadays [4, 5]. Microblading is used for eyebrow restoration, correction, and hair loss [4, 5]. However, microblading can cause scarring and other side effects [5].

Brow lamination is a new technique used to straighten and shape the eyebrows [6]. The first step of brow lamination includes the use of thioglycolic acid (TGA) or ammonium thioglycolate (ATG) also known as thioglycolic acid ammonium salt [7, 8]. These two chemicals are related as ATG can be synthesized by combining TGA with aqueous ammonia [9]. In esthetic formulations, ATG of cosmetic grade generally comprises 60% ATG and up to 2% dithiodiglycolate [9]. Over the years, studies have shown that these chemicals can lead to many side effects affecting the skin, the hair, and the respiratory, reproductive, ocular, and endocrine systems, and that they should not exceed a concentration of 15.2% for use in various hair treatments, including hair straighteners, permanent waves, tonics, and hair dyes [10]. To compare their concentrations since they significantly vary in their molecular weights (MW), an 18% concentration of Ammonium Thioglycolate (MW 109.13) corresponds to approximately 15.2% Thioglycolic Acid (calculated as 18 × 92.12/109.13) [9].

Although the side effects of TGA and ATG on the hair and scalp have been already discussed in research studies, their involvement in the brow lamination technique—in which these chemicals are applied on the thinner skin on the forehead—is still not documented. To our knowledge, this is the first review that aims to highlight the possible outcomes of brow lamination.

## 2 Brow Lamination and Skin

### 2.1 The Skin's Absorption of Ammonium Thioglycolate and Thioglycolic Acid

The skin is the organ most commonly exposed to chemical compounds, such as ATG and TGA, particularly in the case of brow lamination [11]. The absorption properties of compounds, specifically those of these two, through the skin's three layers, take into account various factors [12]. The physicochemical qualities of the latter and the complex structure of the skin have an intricate relationship with dermal absorption [12]. The hydrophilic nature of these compounds allows them to penetrate and reach the bloodstream via two main routes upon skin contact: the intracellular route and the transappendageal route [13, 14, 15]. The intracellular pathway entails penetration through the stratum corneum's corneocytes while the transappendageal pathway allows molecules to pass through sebaceous glands and hair follicles [16, 17].

With the stratum corneum being the outermost layer of the epidermis, its variation in thickness and solubility properties across different areas of the skin surface plays an instrumental role in transdermal permeation [18]. In particular, facial skin generally features a stratum corneum thinner than that of the trunk and limbs which may explain a higher absorption rate of ATG and TGA on the forehead area [19]. Moreover, various studies have demonstrated their absorption is possible through uninjured skin as systemic manifestations arise upon topical application of permanent waving solutions, showing to be chemicals of high toxicity [9, 20, 21]. Furthermore, a large study reported that after the application of 250 μL of thioglycolates to 1–2 cm^2^ of skin surface, an average of 36 μg/cm^2^ permeated the skin after 8 h, and 10 275 μg/cm^2^ was absorbed after 48 h [9]. After dermal application of TGA, 5%–8% was excreted in urine after 1 h, and 30%–50% after 5 h [22]. Therefore, we can infer that the latter may apply to brow lamination which essentially contains ATG and TGA. Overall, having a clear understanding of the dermal absorption of these two substances is crucial for assessing their safety and efficacy in cosmetic applications. Hence, there is great value in doing comprehensive studies on skin absorption mechanisms while factoring in skin physiology and chemical characteristics.

### 2.2 The Potential Impact of Brow Lamination on the Skin

Since skin disorders—essentially allergic contact dermatitis (ACD)—constitute a remarkable public health burden, it is important to focus on whether brow lamination can result in ACD due to its main chemicals, TGA or ATG [11]. ACD is a multifactorial disease influenced by both genetic and environmental factors [11]. ACD, a type IV hypersensitivity reaction, comprises two phases: sensitization and elicitation [23]. In the first phase, the sensitization one—ATG or TGA—infiltrates the stratum corneum [11]. The thioglycolate compound attaches to skin proteins forming an antigen complex [24, 25, 26]. This procedure triggers an innate immune reaction releasing numerous cytokines such as interleukins (IL), including IL‐1α, IL‐1β, IL‐8, IL‐18, as well as tumor necrosis factor‐α (TNF‐α), and granulocyte‐macrophage colony‐stimulating factor (GM‐CSF) [23]. Following this reaction, the ATG or TGA is removed by antigen‐presenting cells (APCs) such as Langerhans cells and dermal dendritic cells [23]. The Langerhans cells travel to the regional lymph nodes where they encounter T lymphocytes successively becoming antigen‐specific T lymphocytes [27]. Subsequently, the antigen‐specific T cells counting the T helper type 1 (Th1), T helper type 2 (Th2), T helper type 17 (Th17), and T regulatory cells multiply and travel in the blood [23]. Additionally, effector and memory T cells are produced following the contact of naïve T cells with the complex allergen‐major histocompatibility complex (MHC) molecule [23]. The second phase, the elicitation phase, involves the reaction that arises following another contact with the same antigen, after repeating the brow lamination procedure [27].

ATG is also commonly found in occupational contact dermatitis: many studies conducted over the years have demonstrated that ATG found in cosmetic products that are used by hairdressers can lead to ACD [28, 29, 30]. A patch testing study conducted in Japan on 203 patients with suspected ACD caused by hair dyes or perming solutions showed 4.8% positivity for ATG within 2 days of exposure [28]. In another study conducted in Brazil, 17.81% of patients showed sensitization to ATG in cosmetic components [29]. Moreover, another study showed a positivity for ATG in 11% of hairdressers with contact dermatitis [30]. In a 10‐year study, an increase in sensitization to ATG from 2.7% to 12.3% was observed in 300 hairdressers with contact dermatitis [31]. In Poland, 7.1% of hairdressers with contact dermatitis were sensitized to ATG [32]. Not only ATG can induce contact dermatitis in hairdressers, but also in their clients [33]. Hair dying is correlated with the greatest possibility of sensitization among hairdressers' clients leading to ACD [33]. We can conclude that the ATG—which is an essential component of the brow lamination technique—is an allergen that can induce contact dermatitis.

Different histological and clinical manifestations of ATG‐induced dermatitis were highlighted in the literature. Many TGA‐related ACD patients showed chronic parafollicular inflammatory cellular infiltrate with fibrotic dermis on histology [34]. Furthermore, experimental studies demonstrated that sodium thioglycolate has led to epidermal hyperplasia, hyperkeratosis, ulceration, and thickening of the skin [22]. Additionally, TGA also causes inflammation and necrosis: the degree of skin damage varies with the dose and concentration of thioglycolic acid [35]. Moreover, the level of IL‐1α, which is an inflammatory interleukin, increased due to product buildup [36].

According to studies mentioned by Burnett et al., the seriousness of the dermal manifestations relies on thioglycolates concentrations and exposure time whereby classifying cold wave products as moderately irritating substances for the skin upon application to rabbit shaved skin due to its composition of ATG. These manifestations range from erythema, inflammation, and contact dermatitis [9]. A research study was carried out asses the degree of correlation between 99% pure TGA and dermatitis resulting in its classification as “causing severe burns” as it may have corrosive properties upon skin contact causing severe burns [9]. In addition, a case study on a hairdresser's skin condition after exposure to permanent waving solutions with ATG presented with exacerbation of irritant dermatitis on the hands [9]. The latter was depicted as papulovesicular eczema, fissures, erosions, white dermographism, and dry, scaly skin on the upper extremities [9]. However, an acute dermal toxicity with ATG results in slight erythema [9]. Another study showed that most patients with ACD caused by ATG presented with skin dryness and rhagades mainly on the hands [20]. To avoid such dermal toxicities of thioglycolate compounds found in many hair products, Uter et al. discussed a systematic review focusing on the importance of wearing gloves when dealing with ATG and product safety. However, these gloves should be disposed of when done with a client as they have been proven to develop into a reservoir which could pose a threat to the hairdresser upon second use [37]. Hence, one can infer that thioglycolate compounds may be equally toxic when applied directly to the skin and eyebrows in brow lamination techniques.

Similar to hairdressers, beauticians also deal with products that contain thioglycolate compounds like hair dyes, cold permanent wave primary solutions, and shampoo [38]. Among seven beauticians associated with occupational dermatitis that were re‐exposed to ATG, three developed a hypersensitivity reaction that presented within a timeframe of 1 month to a year [38]. As a result, the lack of an instant reaction on product application can be seen as an indication of safety for a client wanting to repeat a brow lamination procedure. Furthermore, it is recommended that beauticians with an individual or familial history of allergic tendency refrain from such an occupation due to the risk of future sensitization [38]. The same can be applied to people opting for brow lamination: those with a family history of atopy can be at higher risk to develop ACD.

On another note, during experiments on guinea pigs, TGA has been shown to stimulate skin histidase activity [9, 39]. Nevertheless, the substrate of this enzyme—Histidine—along with arginine and tryptophan has been shown to strengthen the body's immunological function [40]. In particular, L‐histidine is a fundamental component in the synthesis of proteins and plays an instrumental role in various processes throughout the body such as buffering of protons, adhering to metal ions, fostering erythrocyte production, and modulating histamine signaling pathway [41]. Additionally, L‐histidine contributes to the antioxidant activity of the body, whereby it breaks down free radicals, thus minimizing the adverse reactions caused by oxidative stress [42]. Consequently, this implies that TGA leads to a decrease in histidine levels, hence a decrease in immunological function, which in turn enables oxidative stress to go unopposed, potentially leading to damage [42]. The latter translates into various skin manifestations that range from pigmentations and aging to dysbiosis of the skin microbiota, atopic dermatitis, and possibly melanoma [43, 44, 45]. Reactive oxygen species (ROS) are important in the progression of ACD and acute contact dermatitis [46]. In addition to the reaction initiated by an allergen, ROS enhance costimulatory signals for nuclear factor kappa B (NF‐κB) [43]. NF‐κB regulates the prostaglandin pathway and the expression COX‐2 [43]. Likewise, it has been suggested that there is a relationship between oxidative stress and acne vulgaris—chronic inflammatory dermatological disorder—which is linked to elevated levels of squalene and sebum output [44]. Peroxidation of the lipids present in the sebum takes place when the body's antioxidant defense is compromised, provoking an inflammatory reaction [45]. Accordingly, the use of antioxidants may compensate for the deficiency and neutralize the activity of ROS. Likewise, it has been suggested that there is a relationship between oxidative stress and a chronic inflammatory dermatological disorder, the acne vulgaris which is linked to elevated levels of squalene and sebum output [44]. Peroxidation of the lipids present in the sebum takes place when the body's antioxidant defense is compromised, provoking an inflammatory reaction [45]. Accordingly, the use of antioxidants may compensate for the deficiency and neutralize the activity of ROS [47].

Moreover, studies reveal that around 70% of hairdressers develop occupational skin diseases—mostly hand dermatitis—during their professional lives with wet labor being among the primary contributors to sensitization [37, 48]. Continuous contact with water and moist environment weakens one's epidermal barrier and causes irritation, facilitating allergen's penetration and ultimately leads to the manifestation of contact dermatitis [37]. The initial stages of skin irritation and sensitive skin predispose the individual to develop an allergic reaction if triggered by a substance upon contact creating an inflammatory environment that fosters sensitivity [37, 49]. Consequently, one can postulate that the aforementioned effects and barrier disruption would be applicable for eyebrow‐water contact with subsequent irritation. We can conclude that the TGA present in the brow lamination technique can act on oxidative metabolism, thus leading to skin pigmentations and aging.

**FIGURE 1:**
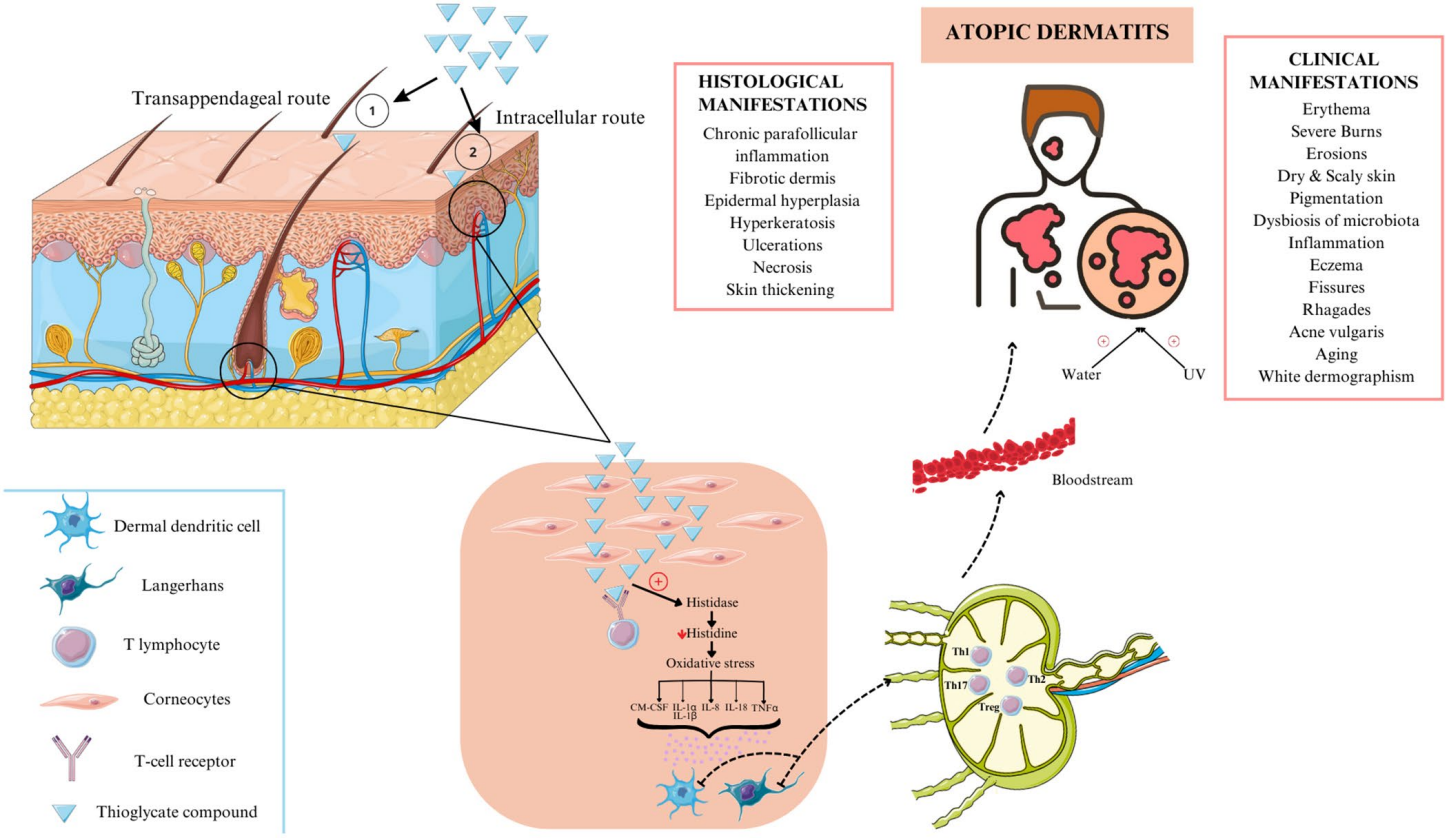
The absorption of thioglycolate compounds with their subsequent effect at the level of the skin.

It is also important to discuss the phototoxicity in people trying brow lamination. There is an intricate relationship between ROS and photoaging; Ultraviolet A (UVA) and ultraviolet B (UVB) both stimulate nicotinamide adenine dinucleotide phosphate (NADPH) oxidase and mitochondrial electron transport chain (ETC) or advanced glycation products, respectively, leading to the synthesis of superoxide radicals (O2^−^), while only UVA reacts with riboflavin and porphyrin—endogenous chromophores—resulting in the production of singlet oxygen (1O2) [50, 51, 52]. Hence, in hope of limiting skin aging effects and improving the body's defense against free radicals, the use of antioxidants, like ascorbic acid, tocopherols, and polyphenols may prove to be beneficial [50]. Thus, it is important to shed light on the potential sun‐induced irritation after the application of thioglycolates compounds. Figure 1 provides a summary of the transdermal absorption of thioglycolate compounds with their respective effects at the level of the skin.

## 3 Brow Lamination and the Structure of Eyebrow Hair

### 3.1 Structure of Eyebrow Hair

**FIGURE 2:**
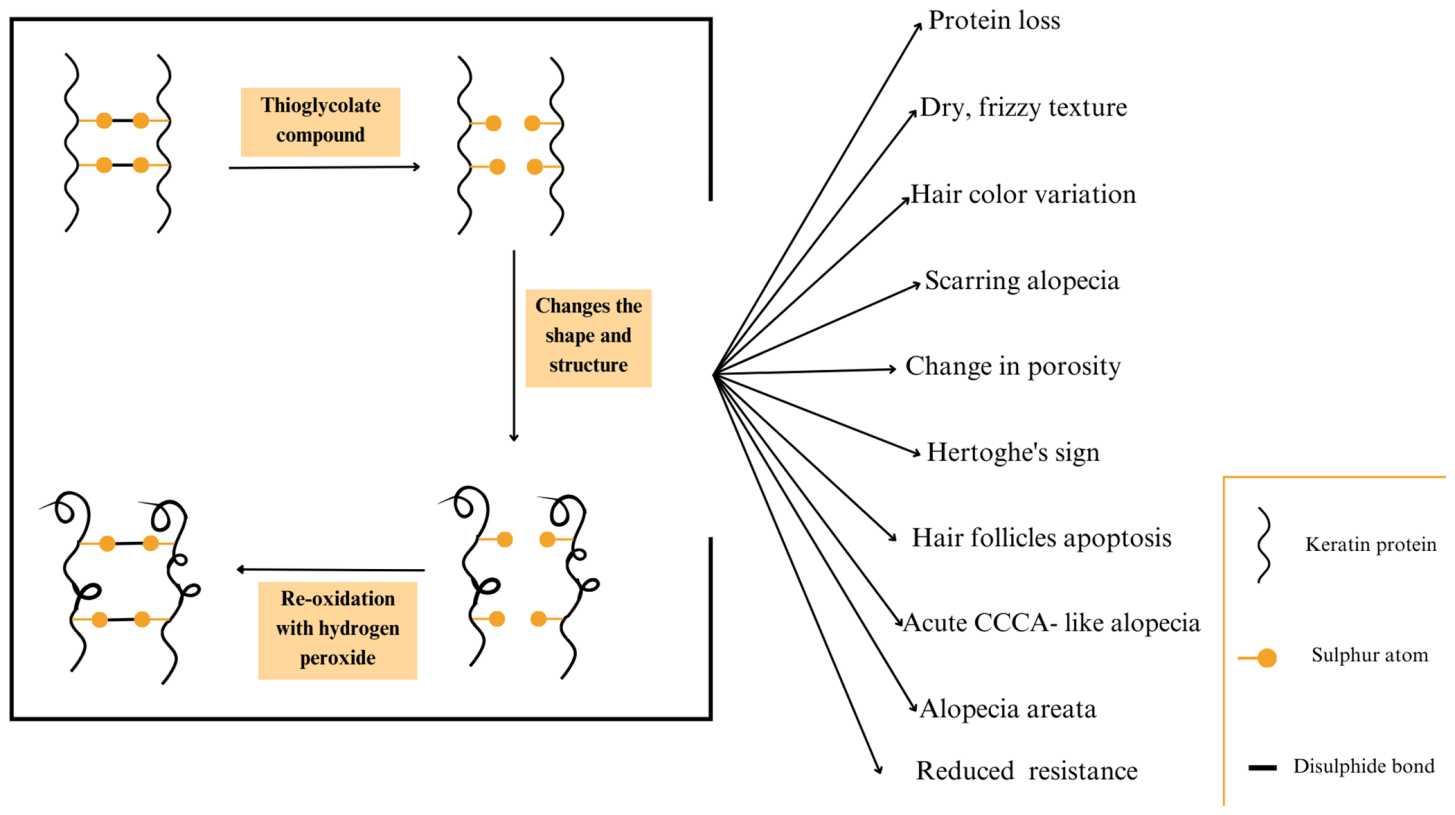
The steps involved in the brow lamination technique.

The hair follicle contains epithelial keratinocytes in the outer root sheath (ORS) and mesenchymal cells in the dermal papilla and dermal sheath, which work together to regulate hair growth. Hair is mainly composed of keratin (about 90%) with a small amount of lipids (1%–9%) (Figure 2) [51, 52]. Keratin, rich in cysteine, forms the hair's structure through disulfide bonds that influence its strength, flexibility, and shape [52]. Hair follicles are crucial in skin and follicle regeneration through a self‐renewing system of epithelial stem cells located in the bulge of the ORS [51, 52]. These stem cells can proliferate and differentiate, aiding in the regeneration and maintenance of skin and hair follicles [51]. In understanding the intricate cellular composition of hair follicles and their crucial role in hair growth and regeneration, it becomes apparent how external factors, such as chemicals found in brow lamination treatments, can disrupt this delicate balance, potentially leading to adverse effects on hair health (Figure 2) [10]. TGA and its derivative, ATG are frequently employed in hair straightening procedures, including perms and relaxers, where they serve as essential reducing agents [10]. According to Zabashta and colleagues' research in 2012, TGA molecules primarily penetrate the disordered regions within the hair fibrils, which are the favored sites for disulfide bond breakage and reformation (Figure 2) [53].

### 3.2 The Effect of Brow Lamination on Eyebrow Hair

Given the importance of disulfide bonds in hair structure and properties, manipulation of these bonds through chemical treatments used in brow lamination can lead to significant effects on hair health (Figure 2) [10]. Research has greatly improved the understanding of how different chemical and physical treatments affect hair morphology and induce specific types of damage (Figure 2) [10]. The chemical treatment allows the hair keratin fibers to undergo mainly two different processes: the reduction process which involves the disconnection of the disulfide (–SS–) groups and the re‐oxidation process to reconnect the groups using the hydrogen peroxide (Figure 2) [10, 54]. The reduction process with TGA, as observed in the study conducted by Akio Kuzuhara published in 2015, led to a remarkable decrease in the conformations of –SS– groups in human hair—the gauche‐gauche‐gauche (GGG) and gauche‐gauche‐trans (GGT) forms—which resulted in the conversion of the cleaved disulfide bonds into cystic acid, signifying a change that weakens the hair and alters its texture [54].

The mechanism of action of ATG and TGA revolves around the alterations of these disulfide bonds within the keratin structure of hair, thereby affecting the hair's configuration (Figure 2) [54]. As ATG penetrates the hair shaft, it promotes the conversion of the existing disulfide bonds (S‐S) in the keratin proteins to sulfhydryl (SH) groups [54]. This conversion process breaks the strong disulfide bonds that provide structure and strength to the hair, allowing the keratin proteins to relax which becomes easier to manipulate into a new shape or configuration (Figure 2) [10]. After the hair has been reshaped, the free sulfhydryl groups are re‐oxidized to reform the disulfide bonds in the desired hair configuration (Figure 2) [54]. This reformation of disulfide bonds locks the hair into its new shape, providing a permanent change to the hair structure (Figure 2) [10, 54]. This disruption of disulfide bonds and reformation of new bonds weaken the overall structure of hair leading to protein loss and reduced mechanical resistance (Figure 2) [10, 54].

In addition, alterations in the disulfide bonds and molecular structure of the hair caused by TGA can increase the porosity of the hair shaft (Figure 2) [54]. High porosity makes the hair shaft predisposed to absorbing and losing moisture which can contribute to dryness and frizz, and a rough texture, making the hair more vulnerable to environmental damage (Figure 2) [54]. A study conducted by Bloch et al. in 2019 concluded that certain types of hair treated with ATG, particularly dark brown and Afro‐ethnic hair, showed increased color variation (Figure 2). This change in color was attributed to the process of opening the hair cuticles during straightening, along with the use of hydrogen peroxide as a neutralizing agent [10]. In essence, these processes can lead to depigmentation of the hair due to the oxidation of melanin granules, resulting in fading or lightening of the hair color [10].

Several studies have delved into the intricate association between chemical hair treatments and their impact on both hair health and scalp condition, particularly focusing on the modulation of interleukin‐alpha (IL‐α) cytokine levels [34, 36]. These studies have illuminated a complex interplay wherein certain chemical hair treatments may lead to an exacerbation of inflammation via the upregulation of IL‐α cytokines [36]. This increase in inflammatory signaling pathways can contribute to diverse adverse effects on the hair and scalp, ranging from heightened sensitivity and irritation to more severe conditions such as dermatitis or alopecia (Figure 2) [34]. Specifically, the findings highlight that the use of strong alkaline compounds found in relaxers, notably those containing ATG, can trigger chemical burns, hair loss, and even severe conditions like chemical misuse‐induced scarring alopecia and acute central centrifugal cicatricial alopecia (CCCA)‐like scarring alopecia (Figure 2) [36].

On the other side, alopecia areata (AA) is an autoimmune non‐scarring condition marked by the sudden loss of eyebrows resulting from the immune system's mistaken attack on hair follicles, which could also be a consideration following brow lamination (Figure 2) [1, 55]. Recent findings revealed elevated levels of oxidative stress index (OSI) and total oxidant capacity (TOC), alongside a decrease in total antioxidant capacity (TAC) among AA patients [56]. The diminished TAC compromises the body's ability to neutralize the ROS molecules and counteract the oxidative stress [56]. This imbalance may heighten oxidative damage within the hair follicles, potentially triggering the apoptosis of hair follicle cells and subsequent hair loss [56]. AA can be a manifestation in people who have done brow lamination due to the effect of thioglycolates compounds on this molecular metabolism.

Inflammation, and the release of pro‐inflammatory cytokines triggered by chemical hair treatments or other factors, can disrupt metabolic processes in the body, including fatty acid oxidation [36]. A study reported that TGA inhibited fatty acid oxidation, leading to a decrease in β‐hydroxybutyrate (βHB) levels in the plasma [9]. This disruption can lead to a decrease in βHB and its derivative, d‐3HB [9]. D‐3HB has been implicated in various physiological processes, including the promotion of cell growth and proliferation [57]. Therefore, decreased d‐3HB levels due to inflammation‐induced alterations in βHB metabolism may compromise the health and function of hair follicles, potentially contributing to hair loss (Figure 2) [57].

Constant scratching and rubbing, triggered by dermatitis caused by the chemical treatments used in brow lamination, can lead to a clinical feature known as Hertoghe's sign or Queen Anne's sign, characterized by the loss of the outer lateral third of the eyebrows (Figure 2) [55].

## 4 The Systemic Effects That the Brow Lamination Technique Might Have

### 4.1 The Potential Impact on Fertility Due to This Technique

Thioglycolates are chemicals that can disrupt hormonal balance in the body, potentially affecting reproductive function [21, 58, 59]. While direct evidence on the reproductive effects of thioglycolate exposure is lacking, some studies have shown reproductive toxicity, including effects on fertility and fetal development in animal models [21, 58, 59].

A meta‐analysis conducted by Kim et al., in 2016, revealed a significantly increased risk of infertility (*p* = 0.1204), fetal death (0.2594), preterm delivery (*p* = 0.5065), small for gestational age (*p* < 0.0001), and low birth weight (*p* = 0.0007) among hairdressers and cosmetologists. The results of this meta‐analysis showed that there is a significant increase in the risk of reproductive disorders among hairdressers and cosmetologists, compared to the general population [58]. Other studies were conducted by Wang et al., in 2011, and Xia et al., in 2011, showing the effect of TGA on parthenogenetic activation of xenopus oocytes, as well as on in vivo oocytes maturation in mice, respectively. They both showed that TGA treatments significantly decreased the activation rate of xenopus oocytes, darkened the animal pole of oocytes gradually, provoked the failure of formation of the female pronucleus, and disturbed the estrous cycle, as well as the structure of testes and ovaries of the rats [21, 59]. In addition, TGA treatment resulted in disturbance in the spindle formation, and chromosome alignment [21, 59].

Another study was performed by Gan et al., in 2003 showing the effect of TGA on menstruation. The results were as follows: disorder of menstrual cycle (*p* = 0.055), disorder of menstrual period (*p* = 0.131), menorrhalgia (*p* = 0.394), and menoxenia (*p* = 0.043) [20]. This emphasizes the hypothesis that TGA negatively affects the menstrual cycle.

After topical administration of a related compound to thioglycolates, the sodium thioglycolate at doses of 50, 100, and 200 mg/kg/day from gestational days 6 to 19 in rats, there was 1 reported maternal death at 200 mg/kg/day, which stands with the fact that TGA is a toxic substance especially for in pregnancy [9]. A study done in 2022 assessed whether the hairdressing occupation is still significantly associated with adverse effects on reproduction, through analyzing six interventional studies conducted from 1995 onwards [60]. These studies investigated outcomes including menstrual disorders, congenital malformations, fetal loss, small‐for‐gestational‐age children, and preterm delivery [60]. Overall, none of the studies showed a significantly increased risk in hairdressers for these outcomes [60]. However, one study from the United States found a borderline significance for congenital malformations, specifically ventricular septal defects in newborns of fathers employed in hairdressing [60]. Additionally, another study found some indices of poor neonatal or maternal health associated with maternal occupation as a hairdresser, although these differences were not significant when compared with a population‐based control group [60]. Regarding infertility, a study found no significant association between various types of female infertility and hairdressing occupation [60]. The overall findings suggest that there is no clear indication that working in the hairdressing industry is still associated with adverse effects on reproduction. However, further research is needed to better understand the potential risks associated with specific hairdressing tasks and occupational exposures.

### 4.2 The Impact of Brow Lamination on the Eyes

Increased evidence suggests that following a direct contact of thioglycolates compounds, the ocular apparatus may be affected. According to a survey‐based study on the health effects of permanent waving solution containing TGA, most hairdressers reported experiencing headaches, dizziness, and dryness in the eyes when applying TGA solution [20]. Hair products that contain TGA, along with its salts and esters, can cause local irritation when they come into contact with the eyes [60].

A study showed that rabbits injected with ATG 17.5% in their eye developed conjunctival redness [61]. In the same study, with rabbits' eyes too, they injected TGA 22%, which also resulted in ocular erythema [61]. This study shows that both ATG and TGA have an irritating effect on the eyes and can cause conjunctival redness [61].

Another study conducted by Burnett et al. in 2009 showed that transient conjunctival redness was observed in the eyes of rabbits after instillation of a cold wave product containing 17.5% ATG. Minimal ocular irritation was also observed in rabbits after instillation of a commercial acid wave containing 22.0% TGA [9].

These studies highlight the fact that thioglycolate causes eyes' dryness, irritation, and redness that can be really disturbing to the patient.

### 4.3 The Consequences of the Lamination Technique on the Respiratory System

While ATG has a major effect on the skin when in contact with it, it can also be inhaled in aerosolized forms causing respiratory irritation and potential toxicity [8]. The mechanism of respiratory irritation involves the release of hydrogen sulfide gas upon contact with moisture in the respiratory tract [62]. The latter can irritate the mucous membranes lining the respiratory passages, leading to symptoms such as coughing, throat irritation, shortness of breath, or even respiratory distress with prolonged exposure [62].

In a study conducted by Liebert et al., in 1991, 14 asthmatic patients inhaled mists of ATG dilutions. After exposure, they exhibited signs and symptoms such as asthmatic breathing, an uncontrollable paroxysmal cough, pharyngeal irritation, and blocked nasal passages or nasal drip [61]. However, non‐asthmatic control patients did not show any positive reactions to the test substance [61]. Following an acute inhalation of a liquid droplet aerosol containing aqueous ATG (60% TGA) in rats for 1 h, findings showed that one of the rats died, and a few experienced a respiratory distress, with no signs observed beyond 24 h post‐exposure [9]. Necropsy revealed minor pulmonary abnormalities [9]. Nasal provocation tests were conducted on hairdressers with occupational rhinitis. Out of 31 patients, one responded positively to a 0.6% ATG solution [9]. We can conclude that thioglycolates compounds can lead to respiratory abnormalities, even at very low concentrations.

Additionally, the effect of another compound on the respiratory system through hydrogen sulfide was highlighted in the literature. A study concluded that the pulmonary excretion of hydrogen sulfide was not noted up to 10 h after intra‐pulmonary injection of a rat with 150 mg/kg of a thioglycolate derivative, the sodium thioglycolate [9]. As much as hydrogen sulfide plays an important role as a gaseous transmitter involved in the control of physiological processes; at high concentrations, it interferes with cellular respiration by inhibiting cytochrome c oxidase in mitochondria, which disrupts the electron transport chain and impairs the body's ability to use oxygen effectively [9]. Excreting hydrogen sulfide is crucial to avoid its toxic effects because its high dose can lead to hypoxia and cellular damage [9].

### 4.4 The Role That Brow Lamination Can Play in Thyroid Dysfunction

While there's limited research on TGA‐specific effects on thyroid function, some studies suggest that certain chemicals or sulfur‐containing compounds found in hair products could potentially disrupt endocrine function, including thyroid hormone regulation [63]. A study showed minimal to slight hyperplasia of the thyroid gland and this could lead to hyperthyroidisms or less commonly hypothyroidism [63].

**FIGURE 3:**
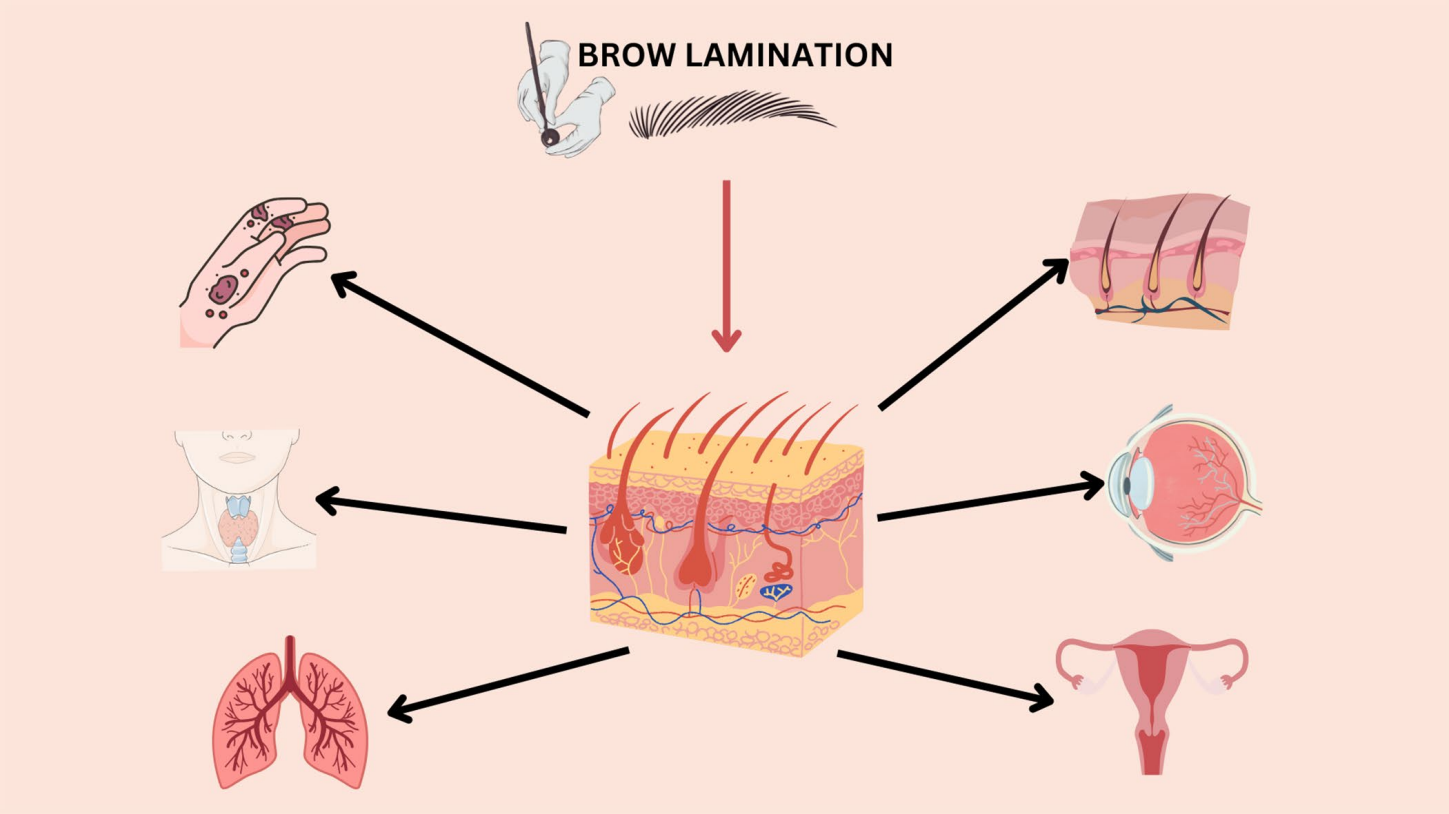
The effect of thioglycolate compounds on the different organ systems.

Hypothyroidism may manifest with a distinctive symptom known as Hertoghe sign or Queen Anne's sign, characterized by the loss of the outer third of the eyebrow. This is a recognizable but not exclusive indication of hypothyroidism [1]. Figure 3 summarizes the side effects of thioglycolate compounds at the level of different organ systems: reproductive, endocrine, ocular, and respiratory systems.

## 5 Conclusion and Future Perspectives

Research has shown that ATG and TGA—used in the first step of brow lamination—have various side effects on the skin, hair, fertility, eyes, respiratory system, and thyroid gland. Therefore, major side effects of brow lamination can include ACD, alterations in the shape, structure, and pigmentation of the hair eyebrow, as well as many other systemic consequences. For this reason, individuals must be aware of the risks posed by these chemicals, the importance of undergoing further testing, including patch and prick tests, and the significance of regulating product consumption. Additional research is crucial to explore potential long‐term side effects other than ACD such as skin cancer. Consequently, different studies and, more importantly, clinical trials should be conducted to rule out the potential incidence of skin cancer among consumers. Until proven otherwise, consumers should resort to other proven harmless alternatives. Finally, present restrictions and regulations must be modified and improved to guarantee consumer safety. Following the recommended usage of such chemicals in regard to careful application, timing, and neutralization is essential to minimize damage and ensure the desired results.

## Author Contributions

L.G. performed the conceptualization, writing the original draft, writing review, and visualization. S.C., A.I., K.M.K., K.K.K. performed the writing the original draft, writing review, and editing and visualization. M.M. performed the writing review and correction.

## Ethics Statement

The authors have nothing to report.

## Consent

The authors have nothing to report.

## Conflicts of Interest

The authors declare no conflicts of interest.

## Data Availability

The data supporting the findings of this study are derived from publicly available articles on PubMed. The full list of articles used in this study is provided in the reference list. These articles can be accessed via PubMed by searching the relevant DOIs or titles included in the references.

## References

[1] B. Nguyen , J. K. Hu , and A. Tosti , “Eyebrow and Eyelash Alopecia: A Clinical Review,” American Journal of Clinical Dermatology 24, no. 1 (2023): 55–67. 36183302 10.1007/s40257-022-00729-5 PMC9870835

[2] J. P. Gunter and S. D. Antrobus , “Aesthetic Analysis of the Eyebrows,” Plastic and Reconstructive Surgery 99, no. 7 (1997): 1808–1816. 9180703 10.1097/00006534-199706000-00002

[3] H. Kim , J. Jung , S. W. Choi , C. W. Yun , and W. Lee , “Eyebrow Lifting Using Multidirectional Absorbable Thread,” Journal of Cosmetic Dermatology 22, no. 10 (2023): 2780–2784. 37060183 10.1111/jocd.15771

[4] A. Bared , “What's New in Facial Hair Transplantation?: Effective Techniques for Beard and Eyebrow Transplantation,” Facial Plastic Surgery Clinics of North America 27, no. 3 (2019): 379–384. 31280852 10.1016/j.fsc.2019.04.003

[5] D. Hvas and J. Serup , “Microblading Technique for Tattooing of “Hairstrokes” That Simulate Natural Hair: Eyebrow Tattooing and Correction of Medical Conditions,” Current Problems in Dermatology 56 (2022): 141–154. 37263228 10.1159/000529810

[6] “What Is Brow Lamination—How Does Brow Lamination Treatment Work,” 2024, https://www.townandcountrymag.com/style/beauty‐products/a38083854/what‐is‐brow‐lamination/.

[7] Nylon , “Brow Lamination Is In Right Now — But Is It Worth It?,” 2020, https://www.nylon.com/what‐is‐brow‐lamination‐explainer.

[8] PubChem , “Ammonium Thioglycolate,” 2024, https://pubchem.ncbi.nlm.nih.gov/compound/21534.

[9] C. L. Burnett , W. F. Bergfeld , D. V. Belsito , et al., “Final Amended Report on the Safety Assessment of Ammonium Thioglycolate, Butyl Thioglycolate, Calcium Thioglycolate, Ethanolamine Thioglycolate, Ethyl Thioglycolate, Glyceryl Thioglycolate, Isooctyl Thioglycolate, Isopropyl Thioglycolate, Magnesium Thioglycolate, Methyl Thioglycolate, Potassium Thioglycolate, Sodium Thioglycolate, and Thioglycolic Acid,” International Journal of Toxicology 28, no. 4 Suppl (2009): 68–133. 19636068 10.1177/1091581809339890

[10] L. D. Bloch , A. M. Goshiyama , M. F. Dario , et al., “Chemical and Physical Treatments Damage Caucasian and Afro‐Ethnic Hair Fibre: Analytical and Image Assays,” Journal of the European Academy of Dermatology and Venereology 33, no. 11 (2019): 2158–2167. 31237371 10.1111/jdv.15761

[11] “Allergic Contact Dermatitis: From Pathophysiology to Development of New Preventive Strategies—ScienceDirect,” 2024, https://www.sciencedirect.com/science/article/abs/pii/S1043661820315905. 10.1016/j.phrs.2020.105282 33161140

[12] “Molecules | Free Full‐Text | Prodrug Strategies for Enhancing the Percutaneous Absorption of Drugs,” 2024, https://www.mdpi.com/1420‐3049/19/12/20780. 10.3390/molecules191220780 PMC6271867 25514222

[13] D. D. N'Da , “Prodrug Strategies for Enhancing the Percutaneous Absorption of Drugs,” Molecules 19, no. 12 (2014): 20780–20807. 25514222 10.3390/molecules191220780 PMC6271867

[14] Y. Fan , L. Lund , Q. Shao , Y. Q. Gao , and F. M. Raushel , “A Combined Theoretical and Experimental Study of the Ammonia Tunnel in Carbamoyl Phosphate Synthetase,” Journal of the American Chemical Society 131, no. 29 (2009): 10211–10219. 19569682 10.1021/ja902557r PMC2748306

[15] B. A. Koeneman , Y. Zhang , K. Hristovski , et al., “Experimental Approach for an In Vitro Toxicity Assay With Non‐Aggregated Quantum Dots,” Toxicology In Vitro 23, no. 5 (2009): 955–962. 19465109 10.1016/j.tiv.2009.05.007

[16] A. Williams , “Transdermal and Topical Drug Delivery,” Transdermal and Topical Drug Delivery: From Theory to Clinical Practice 4, no. 1 (2003): 178–187.

[17] “Novel Mechanisms and Devices to Enable Successful Transdermal Drug Delivery – ScienceDirect,” 2024, https://www.sciencedirect.com/science/article/abs/pii/S0928098701001671?via%3Dihub. 10.1016/s0928-0987(01)00167-1 11500256

[18] D. Howes , R. Guy , J. Hadgraft , et al., “Methods for Assessing Percutaneous Absorption: The Report and Recommendations of ECVAM Workshop 131,2,” 1996, 10.1177/026119299602400111.

[19] H. Tagami , “Location‐Related Differences in Structure and Function of the Stratum Corneum With Special Emphasis on Those of the Facial Skin,” International Journal of Cosmetic Science 30, no. 6 (2008): 413–434. 19099543 10.1111/j.1468-2494.2008.00459.x

[20] H. Gan , X. Meng , C. Song , and B. Li , “A Survey on Health Effects in a Human Population Exposed to Permanent‐Waving Solution Containing Thioglycolic Acid,” Journal of Occupational Health 45, no. 6 (2003): 400–404. 14676420 10.1539/joh.45.400

[21] Z. Wang , X. Ren , D. Wang , Y. Guan , and L. Xia , “Effects of Thioglycolic Acid on Parthenogenetic Activation of Xenopus Oocytes,” PLoS One 6, no. 1 (2011): e16220. 21297954 10.1371/journal.pone.0016220 PMC3031513

[22] N. T. Program , N. T. Program , M. Mercado‐Feliciano , et al., NTP Technical Report on the Toxicity Studies of Sodium Thioglycolate (CASRN 367–51‐1) Administered Dermally to F344/N Rats and B6C3F1/N Mice (Research Triangle Park, NC: National Toxicology Program, 2016). 10.22427/NTP-TOX-80 PMC8040344 33530656

[23] M. Tramontana , K. Hansel , L. Bianchi , C. Sensini , N. Malatesta , and L. Stingeni , “Advancing the Understanding of Allergic Contact Dermatitis: From Pathophysiology to Novel Therapeutic Approaches,” Frontiers in Medicine 10 (2023): 1184289, 10.3389/fmed.2023.1184289. 37283623 PMC10239928

[24] R. P. Usatine and M. Riojas , “Diagnosis and Management of Contact Dermatitis,” American Family Physician 82, no. 3 (2010): 249–255. 20672788

[25] L. Kostner , F. Anzengruber , C. Guillod , M. Recher , P. Schmid‐Grendelmeier , and A. A. Navarini , “Allergic Contact Dermatitis,” Immunology and Allergy Clinics of North America 37, no. 1 (2017): 141–152. 27886903 10.1016/j.iac.2016.08.014

[26] S. Nassau and L. Fonacier , “Allergic Contact Dermatitis,” Medical Clinics of North America 104, no. 1 (2020): 61–76. 31757238 10.1016/j.mcna.2019.08.012

[27] P. B. Murphy , A. R. Atwater , and M. Mueller , “Allergic Contact Dermatitis,” in StatPearls (Treasure Island (FL): StatPearls Publishing, 2024). 33760443

[28] A. Ito , K. Nishioka , H. Kanto , et al., “A Multi‐Institutional Joint Study of Contact Dermatitis Related to Hair Colouring and Perming Agents in japan,” Contact Dermatitis 77, no. 1 (2017): 42–48. 28425114 10.1111/cod.12783

[29] E. A. Silva , M. R. M. Bosco , and É. Mozer , “Study of the Frequency of Allergens in Cosmetics Components in Patients With Suspected Allergic Contact Dermatitis,” Anais Brasileiros de Dermatologia 87 (2012): 263–268. 22570031 10.1590/s0365-05962012000200011

[30] A. Katsarou , B. Koufou , K. Takou , D. Kalogeromitros , G. Papanayiotou , and A. Vareltzidis , “Patch Test Results in Hairdressers With Contact Dermatitis in Greece (1985–1994),” Contact Dermatitis 33, no. 5 (1995): 347–348. 8565491 10.1111/j.1600-0536.1995.tb02050.x

[31] R. Valks , L. Conde‐Salazar , J. Malfeito , and S. Ledo , “Contact Dermatitis in Hairdressers, 10 Years Later: Patch‐Test Results in 300 Hairdressers (1994 to 2003) and Comparison With Previous Study,” Dermatitis 16, no. 1 (2005): 28–31. 15996347

[32] M. Kieć‐Swierczyńska , B. Krecisz , and D. Chomiczewska , “Results of Patch Test in Hairdressers Examined in the Institute of Occupational Medicine in Łódź,” Medycyna Pracy 60, no. 6 (2009): 459–467. 20187494

[33] L. Gurrra , F. Bardazzi , and A. Tosti , “Contact Dermatitis in hairdressers' Clients,” Contact Dermatitis 26, no. 2 (1992): 108–111. 1633700 10.1111/j.1600-0536.1992.tb00893.x

[34] A. G. Nicholson , C. C. Harland , R. H. Bull , P. S. Morttmhr , and M. G. Cook , “Chemically Induced Cosmetic Alopecia,” British Journal of Dermatology 128, no. 5 (1993): 537–541. 8504045 10.1111/j.1365-2133.1993.tb00231.x

[35] L. L. Gershbein , “Percutaneous Toxicity of Thioglycolate Mixtures in Rabbits,” Journal of Pharmaceutical Sciences 68, no. 10 (1979): 1230–1235. 512851 10.1002/jps.2600681009

[36] R. A. Beach , K. A. Wilkinson , F. Gumedze , and N. P. Khumalo , “Baseline Sebum IL‐1α Is Higher Than Expected in Afro‐Textured Hair: A Risk Factor for Hair Loss?,” Journal of Cosmetic Dermatology 11, no. 1 (2012): 9–16. 22360329 10.1111/j.1473-2165.2011.00603.x

[37] W. Uter , J. Strahwald , S. Hallmann , et al., “Systematic Review on Skin Adverse Effects of Important Hazardous Hair Cosmetic Ingredients With a Focus on Hairdressers,” Contact Dermatitis 88, no. 2 (2023): 93–108. 36254351 10.1111/cod.14236

[38] K. Matsunaga , K. Hosokawa , M. Suzuki , Y. Arima , and R. Hayakawa , “Occupational Allergic Contact Dermatitis in Beauticians,” Contact Dermatitis 18, no. 2 (1988): 94–96. 2966708 10.1111/j.1600-0536.1988.tb02747.x

[39] J. H. Anglin , D. H. Jones , A. T. Bever , and M. A. Everett , “The Effect of Ultraviolet Light and Thiol Compounds on Guinea Pig Skin Histidase,” Journal of Investigative Dermatology 46, no. 1 (1966): 34–39. 5905251 10.1038/jid.1966.7

[40] A. A. Tantawy and D. M. Naguib , “Arginine, Histidine and Tryptophan: A New Hope for Cancer Immunotherapy,” PharmaNutrition 1, no. 8 (2019): 100149.

[41] M. Holeček , “Histidine in Health and Disease: Metabolism, Physiological Importance, and Use as a Supplement,” Nutrients 12, no. 3 (2020): 848. 32235743 10.3390/nu12030848 PMC7146355

[42] G. Aguirre‐Cruz , A. León‐López , V. Cruz‐Gómez , R. Jiménez‐Alvarado , and G. Aguirre‐Álvarez , “Collagen Hydrolysates for Skin Protection: Oral Administration and Topical Formulation,” Antioxidants (Basel) 9, no. 2 (2020): 181. 32098294 10.3390/antiox9020181 PMC7070905

[43] S. P. Faux and P. J. Howden , “Possible Role of Lipid Peroxidation in the Induction of NF‐Kappa B and AP‐1 in RFL‐6 Cells by Crocidolite Asbestos: Evidence Following Protection by Vitamin E,” Environmental Health Perspectives 105 Suppl 5, no. Suppl 5 (1997): 1127–1130. 9400711 10.1289/ehp.97105s51127 PMC1470127

[44] G. L. Popa , C. I. Mitran , M. I. Mitran , et al., “Markers of Oxidative Stress in Patients With Acne: A Literature Review,” Life 13, no. 7 (2023): 1433. 37511808 10.3390/life13071433 PMC10381563

[45] M. A. Ibrahim , M. E. Helmy , H. H. Sabry , S. M. Farouk , L. Y. Ebrahim , and E. R. Amer , “Evaluation of Biomarkers of Oxidant–Antioxidant Balance in Patients With Acne Vulgaris,” Journal of the Egyptian Women's Dermatologic Society 12, no. 2 (2015): 136–141.

[46] D. H. Kim , D. Byamba , W. H. Wu , T. G. Kim , and M. G. Lee , “Different Characteristics of Reactive Oxygen Species Production by Human Keratinocyte Cell Line Cells in Response to Allergens and Irritants,” Experimental Dermatology 21, no. 2 (2012): 99–103. 22141451 10.1111/j.1600-0625.2011.01399.x

[47] H. Masaki , Y. Okano , and H. Sakurai , “Generation of Active Oxygen Species From Advanced Glycation End‐Products (AGEs) During Ultraviolet Light A (UVA) Irradiation and a Possible Mechanism for Cell Damaging,” Biochimica et Biophysica Acta 1428, no. 1 (1999): 45–56. 10366759 10.1016/s0304-4165(99)00056-2

[48] C. Symanzik , J. D. Johansen , P. Weinert , et al., “Differences Between Hairdressers and Consumers in Skin Exposure to Hair Cosmetic Products: A Review,” Contact Dermatitis 86, no. 5 (2022): 333–343. 35088418 10.1111/cod.14055

[49] H. J. Schwanitz and W. Uter , “Interdigital Dermatitis: Sentinel Skin Damage in Hairdressers – Schwanitz,” British Journal of Dermatology 142 (2000): 1011–1012, 10.1046/j.1365-2133.2000.03487.x. 10877542

[50] C. Oresajo , S. Pillai , M. Manco , M. Yatskayer , and D. McDaniel , “Antioxidants and the Skin: Understanding Formulation and Efficacy,” Dermatologic Therapy 25, no. 3 (2012): 252–259. 22913443 10.1111/j.1529-8019.2012.01505.x

[51] I. Han , K. J. Shim , J. Y. Kim , et al., “Effect of Poly(3‐Hydroxybutyrate‐Co‐3‐Hydroxyvalerate) Nanofiber Matrices Cocultured With Hair Follicular Epithelial and Dermal Cells for Biological Wound Dressing,” Artificial Organs 31, no. 11 (2007): 801–808. 18001389 10.1111/j.1525-1594.2007.00466.x

[52] M. V. R. Velasco , T. C. de Sá‐Dias , M. F. Dario , et al., “Impact of Acid (“Progressive Brush”) and Alkaline Straightening on the Hair Fiber: Differential Effects on the Cuticle and Cortex Properties,” International Journal of Trichology 14, no. 6 (2022): 197–203. 37034547 10.4103/ijt.ijt_158_20 PMC10075350

[53] Y. F. Zabashta , A. V. Kasprova , S. P. Senchurov , and Y. E. Grabovskii , “The Location of the Thioglycolic Acid Molecules in Intrafibrillar Unordered Areas of the Human Hair Keratin Structure,” International Journal of Cosmetic Science 34, no. 3 (2012): 223–225. 22268367 10.1111/j.1468-2494.2012.00707.x

[54] A. Kuzuhara , “Internal Structural Changes in Keratin Fibres Resulting From Combined Hair Waving and Stress Relaxation Treatments: A Raman Spectroscopic Investigation,” International Journal of Cosmetic Science 38, no. 2 (2016): 201–209. 26383008 10.1111/ics.12278

[55] A. Kumar and K. Karthikeyan , “Madarosis: A Marker of Many Maladies,” International Journal of Trichology 4, no. 1 (2012): 3–18. 22628984 10.4103/0974-7753.96079 PMC3358936

[56] J. Baek and M. G. Lee , “Oxidative Stress and Antioxidant Strategies in Dermatology,” Redox Report 21, no. 4 (2016): 164–169. 26020527 10.1179/1351000215Y.0000000015 PMC8900706

[57] X. Yao and S. Matosevic , “Cryopreservation of NK and T Cells Without DMSO for Adoptive Cell‐Based Immunotherapy,” BioDrugs 35, no. 5 (2021): 529–545. 34427899 10.1007/s40259-021-00494-7 PMC12376086

[58] D. Kim , M. Y. Kang , S. Choi , J. Park , H. J. Lee , and E. A. Kim , “Reproductive Disorders Among Cosmetologists and Hairdressers: A Meta‐Analysis,” International Archives of Occupational and Environmental Health 89 (2016): 739–753. 26821358 10.1007/s00420-016-1112-z PMC4871926

[59] L. Xia , S. Hou , X. Ren , and Z. Wang , “Effects of Thioglycolic Acid on In Vivo Oocytes Maturation in Mice,” PLoS One 6, no. 9 (2011): e23996. 21909408 10.1371/journal.pone.0023996 PMC3164699

[60] Ž. Babić , M. Macan , Z. Franić , et al., “Association of Hairdressing With Cancer and Reproductive Diseases: A Systematic Review,” Journal of Occupational Health 64, no. 1 (2022): e12351. 36017574 10.1002/1348-9585.12351 PMC9411989

[61] Dermatitis® (Mary Ann Liebert, Inc., Publishers, 2024), https://home.liebertpub.com/publications/dermatitis/672/overview.

[62] S. Khattak , Q. Q. Zhang , M. Sarfraz , et al., “The Role of Hydrogen Sulfide in Respiratory Diseases,” Biomolecules 11, no. 5 (2021): 682. 34062820 10.3390/biom11050682 PMC8147381

[63] L. Chaker , A. C. Bianco , J. Jonklaas , and R. P. Peeters , “Hypothyroidism,” Lancet 390, no. 10101 (2017): 1550–1562. 28336049 10.1016/S0140-6736(17)30703-1 PMC6619426

